# Preformulation Studies of Furosemide-Loaded Electrospun Nanofibrous Systems for Buccal Administration

**DOI:** 10.3390/polym9120643

**Published:** 2017-11-25

**Authors:** Andrea Kovács, Balázs Démuth, Andrea Meskó, Romána Zelkó

**Affiliations:** 1 Gedeon Richter Plc., Formulation R&D, Gyömrői Street 19-21, H-1103 Budapest, Hungary; kovacs.andrea@pharma.semmelweis-univ.hu; 2 Department of Organic Chemistry and Technology, Budapest University of Technology and Economics, Budafoki út 8. 3, H-1103 Budapest, Hungary; demuth@oct.bme.hu; 3 University Pharmacy Department of Pharmacy Administration, Semmelweis University, Hőgyes Endre Street 7-9, H-1092 Budapest, Hungary; mesko.attilane@pharma.semmelweis-univ.hu

**Keywords:** furosemide, electrospinning, hydroxypropyl cellulose, poly (vinylpyrrolidone), storage and loss moduli, scanning electron microscopic images

## Abstract

Furosemide loaded electrospun fibers were prepared for buccal administration, with the aim of improving the oral bioavailability of the poorly soluble and permeable crystalline drug, which can be achieved by the increased solubility and by the circumvention of the intensive first pass metabolism. The water soluble hydroxypropyl cellulose (HPC) was chosen as a mucoadhesive polymer. In order to improve the electrospinnability of HPC, poly (vinylpyrrolidone) (PVP) was used. During the experiments, the total polymer concentration was kept constant at 15% (*w*/*w*), and only the ratio of the two polymers (HPC-PVP = 5:5, 6:4, 7:3, 8:2, 9:1) was changed. A combination of rheological measurements with scanning electron microscopic morphological images of electrospun samples was applied for the determination of the optimum composition of the gels for fiber formation. The crystalline–amorphous transition of furosemide was tracked by Fourier transform infrared spectroscopy. A correlation was found between the rheological properties of the polymer solutions and their electrospinnability, and the consequent morphology of the resultant samples. With decreasing HPC ratio of the system, a transition from the spray-dried droplets to the randomly oriented fibrous structures was observed. The results enable the determination of the polymer ratio for the formation of applicable quality of electrospun fibers.

## 1. Introduction

Electrospinning is an emerging technology by which mats of micro- and nanofibers can be produced using an electrostatically driven jet of polymer solution or melt. A wide variety of micro- and nanofibers have been formulated by this process using several natural and synthetic polymers. The use of electrospinning for the fabrication of drug-loaded nanofibrous scaffolds is a promising alternative for developing delivery systems of high efficiency. Many types of drugs can be easily incorporated into electrospun materials. Along with the changes of the morphology, the porosity and the composition of the nanofibers, and the release profile of the incorporated drug can be fine-tuned [1].

Furosemide is used as a loop diuretic in treatment of hypertension, and in renal, cardiac, and hepatic oedematous status. It belongs to the BCS (Biopharmaceutical Classification System) IV class, due to its poor water soluble properties (5–20 μg/mL at pH = 7) and low membrane permeability, and thus, poor bioavailability [2,3,4,5,6,7]. The low and variable absorption from the stomach and upper part of the duodenum in the gastrointestinal tract, and presence of intestinal efflux proteins, make its therapeutic use difficult. The reason of the slight absorption is partly the presence of the intestinal efflux proteins, mentioned previously, and in part the ionization (the ionization is 94.06% at pH = 5 and 99.97% at pH = 7.4). In the ileum, the expression of the MDR1 (multidrug resistance P-glycoprotein; PgP) efflux protein is much more than in the jejunum. Therefore, the absorption in the ileum is 2.5 times slower than in the jejunum [8].

Furosemide is commonly applied in oral and intravenous therapies, but many systemic side effects occur during the traditional administrations, like polyuria, mouth drying, dizziness, and gastric problems [9]. Because of the low oral bioavailability and the slow onset of action (even 60 min) the administration of the conventional tablets is not appropriate in emergency situations [10].

The registered furosemide containing medicines are mostly tablets (40, 80, 500 mg) and injections in Hungary [11]. However, several research groups deal with developing new formulations, owing to the enhancement of the water solubility of the furosemide and its consequent bioavailability. Some of these formulations are fast dissolving tablets [10], supramolecular complexes [4,7], melt extrusion methods [12], micro/nanoparticles for drug delivery [13], mucoadhesive microspheres [3], and halloysites [14]. The primary focus of the recent studies was to improve the solubility and permeability of the BCS IV drugs by the use of special formulation strategies.

An alternative formulation of active ingredients with poor water solubility and low bioavailability, is to make a mucoadhesive buccal drug delivery system. It is also a good opportunity to use the drugs in emergency situations without invasive administration. The transmucosal formulations could be an option in the case of enhancing the bioavailability of the furosemide. Using buccal administration, it can be possible to circumvent the first pass metabolism. As consequence of the rich vascularization of the buccal mucosa, the drug can be easily taken to the systemic circulation. On the other hand, the buccal epithelium has a barrier function, which makes penetration difficult, therefore, it is necessary the use of permeation enhancers during the formulation [15,16,17].

Electrospun nanofibrous drug delivery systems could be promising alternative forms with their advantages, like high porosity, high surface to volume ratio, and the transition of crystalline to amorphous form, thus enhancing solubility [18,19,20].

The aim of the present study was to prepare amorphous furosemide-loaded mucoadhesive fibrous formulations containing solubilizer, which could also act as a penetration enhancer for buccal application. Aqueous solution was used for the electrospinning to generate nanofibers of environmentally friendly conditions, and consequently, there was no need to define the residual solvent content. In order to select the optimum composition, the authors provide detail and adequate characterization of the fibers with rheology, size, morphology, and solid-state characterization. The correlation between the fiber forming ability and the rheological properties of the initial drug-containing HPC-PVP solutions and their electrospinnability, and the consequent morphology of the resultant samples, was also determined.

## 2. Materials and Methods

### 2.1. Materials

**Figure 1:**
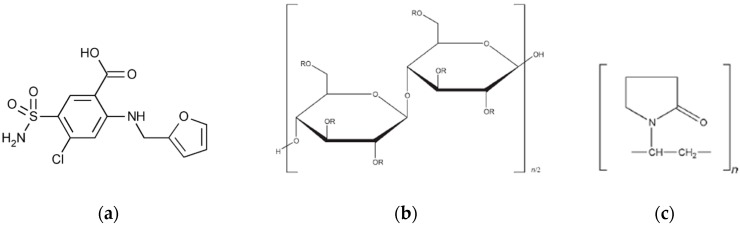
Chemical structure of furosemide (**a**) and the structural units of fiber forming polymers ((**b**) HPC, (**c**) PVP).

Hydroxypropyl cellulose (Klucel EXF Pharm, Ashland, Covington, KY, USA; Mw ~ 80,000, the moles of substitution = 3.8), poly(vinylpyrrolidone) (Kollidon 90 F, BASF, Ludwigshafen, Germany; Mw ~ 1,000,000–1,500,000) as polymers (Figure 1b,c), trolamine (Ph. Eur., Molar Chemicals, Budapest, Hungary) as surfactant, and purified water was used as solvent for the polymers. Furosemide (Ph. Eur., Figure 1a) was used as model drug from the BCS IV class.

### 2.2. Preparation of Furosemide Containing HPC-PVP Gels

The stock solution of furosemide was prepared by adding 0.2 furosemide into a dark beaker, and it was dissolved with 17.2 g purified water in a few minutes under magnetic stirring by adding 0.3 g trolamine as solubilizer. The gels were prepared by using the furosemide solution in each case, and a different added amount of PVP. These compositions were being mixed at room temperature (*T* = 25 °C), until they had reached a totally homogenous condition. The necessary amount of HPC was added in small portions to the prepared drug loading PVP gels during stirring, also at room temperature (*T* = 25 °C) to achieve the homogeneity. The total polymer concentration of the gels was 15 wt %, but the *w*/*w* ratio of the applied two polymers was different. The different HPC-PVP ratios were 5:5, 6:4, 7:3, 8:2, 9:1.

### 2.3. Preparation of the Physical Mixtures

For the fourier transform infrared (FT-IR) measurements the powder physical mixtures of the gel components (containing furosemide, polymers, and surfactant) were prepared and homogenized by stirring for 5 min in a porcelain mortar.

### 2.4. Rheological Properties of the Gels

Kinexus Pro Rheometer (Malvern Instruments Ltd., Malvern, UK) was used for measuring the rheological properties of the prepared gels at room temperature (*T* = 25 ± 0.1 °C). During the measurement, data were registered with software of rSpace for Kinexus Pro 1.3 (Malvern Instruments Ltd., Malvern, UK). Parallel plates geometry (diameter: 50 mm) was applied during the tests; the gap between the plates was 0.0300 mm. Elastic (*G*’) and viscous moduli (*G*”) were determined by oscillatory rheological tests. The oscillatory shear measurements were performed at amplitude within the linear region, which was chosen to 30% within the viscoelastic region, and the frequency was in the range of 0.1–10 s^−1^. Three parallel measurements were done with each gel in both tests.

### 2.5. Electrospinning

The nanofibers were prepared by electrospinning at room temperature (25 °C). The gels were put into syringes, after that, silicon tubes with needles (internal diameter: 1.2) on their ends were connected to the syringes. High voltage was applied (25 kV) at 15 cm collector-needle distance. The flow rate was 0.3 mL/h during the spinning process. The prepared fibers and droplets were dried at room temperature during the electrospinning process.

### 2.6. ATR-FTIR (Attenuated Total Reflectance-Fourier Transform Infrared) Spectroscopy Measurements

The ATR-FTIR spectra of the fibers, physical mixtures and raw substances were measured by using Jasco FT/IR-4200 spectrophotometer (Jasco Inc., Easton, MD, USA), which was equipped with Jasco ATR PRO470-H single reflection accessory. The spectra were collected over a wavenumber range of 4000 and 1800 cm^−1^. After 100 scans, the measurements were evaluated with Spectra Manager-II, Jasco software.

### 2.7. Scanning Electron Microscopy (SEM) Measurements

The morphology was examined by JEOL 6380LVa (JEOL, Tokyo, Japan) scanning electron microscope using secondary electron imaging detection method. The collected samples on aluminum foil were fixed with conductive double-sided carbon adhesive tape, and their surface was covered by sputtered gold before the measurement. Accelerating voltage (15 kV) was applied, and the working distance was 10 mm during the examination.

### 2.8. Statistical Analysis of the Fiber Diameters

Statistical comparisons of samples were conducted using SPSS 20.0 software package (SPSS Inc., Chicago, IL, USA). The SEM images were used for the measurement, and the mean diameters were calculated by using Image J program (https://imagej.nih.gov/nih-image/). Fifty measurements from the fibrous elements were carried out in each SEM image. Only the fibers were examined, and the droplets were not considered for the determination of the average diameter size.

## 3. Results and Discussion

### 3.1. Rheological Properties

**Figure 2:**
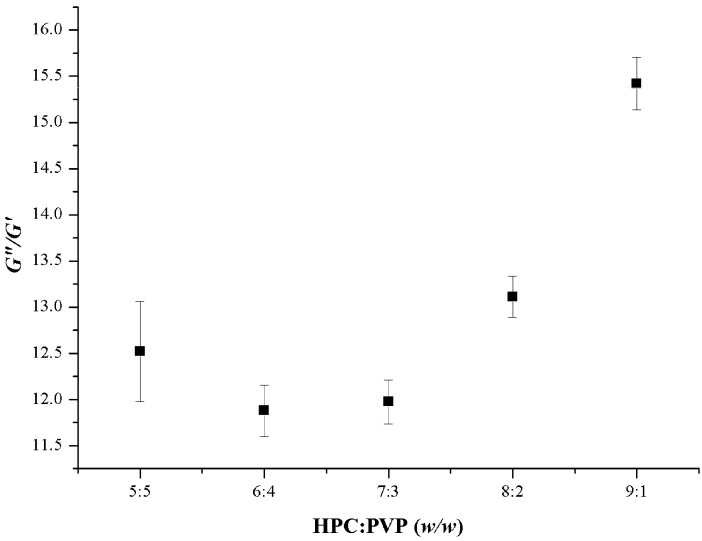
Ratio of loss (*G*”) and storage (*G*’) moduli of furosemide-loaded gels as a function of HPC-PVP mass ratio (oscillatory shear measurements at a frequency 1.995 Hz).

Figure 2 represents the loss (*G*’’)/storage (*G*’) moduli as a function of HPC-PVP ratio. The results indicate, as a function of the SEM images, that there is an optimum viscoelastic behavior of the composite gels, which results in the best fiber forming ability, and thus, more homogeneous randomly-oriented fibrous mats. Similarly to the previous experiences [21], the scanning electron microscopic images of the samples revealed that revealed that there exists a viscoelastic range, which provides an advantageous macrostructural arrangement of the macromolecules for the fiber formation. The latter can be explained by phenomenon that the prevalent viscoelasticity of the sample could be disadvantageous from the point of the formation of homogeneous nanostructured fiber elements, since the electrical forces cannot be effectively transferred to the viscoelastic elongation of the polymer jets in the course of the fiber formation. The most elastic sample (HPC-PVP = 6:4) resulted the most homogenous nanofibrous system.

### 3.2. ATR-FTIR Analysis

**Figure 3:**
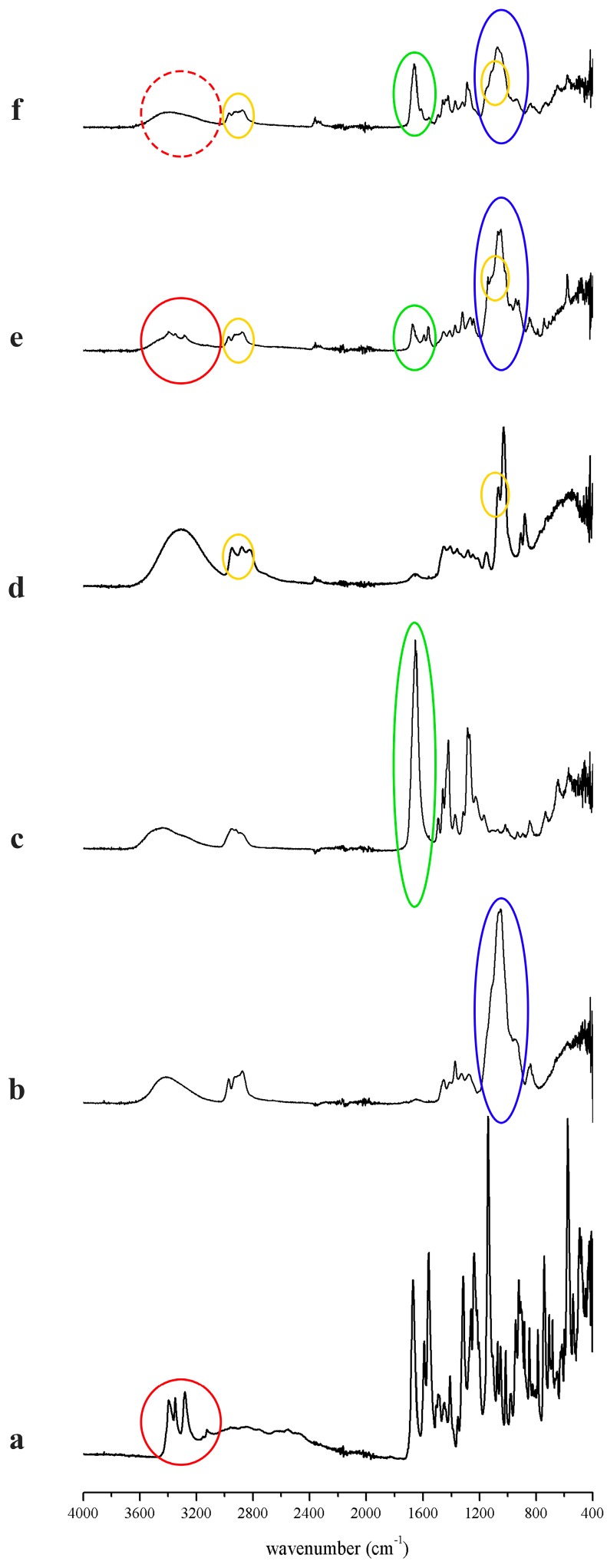
FT-IR spectra of the raw materials, physical mixture and the furosemide containing fibers. (**a**) furosemide; (**b**) HPC EXF; (**c**) PVP 90F; (**d**) trolamine; (**e**) physical mixture (HPC-PVP = 6:4 molar ratio); (**f**) furosemide containing fiber of HPC-PVP = 6:4 molar ratio.

The FT-IR spectra of the raw materials, the physical mixtures and the prepared formulations are presented in the Figure 3. Furosemide has three characteristic peaks in the range of 3400–3200 cm^−1^. The peaks are sulphonamide NH stretching at 3397 cm^−1^ and 3281 cm^−1^, and there is a peak of secondary amine NH stretching at 3349 cm^−1^, which are in good agreement with previous studies [7]. The Figure 3 clearly indicates similar peaks belonging to the sulphonamide NH stretching, and a peak of the secondary amine NH stretching, which are missing in the fibrous sample, meanwhile, these peaks can be distinguished in the physical mixture of the components. In the 1600–500 cm^−1^ “fingerprint region” of the fibers, the characteristic peaks of the fiber-forming polymers (PVP at 1653 cm^−1^ NH bending, HPC at 1100 cm^−1^ C–O stretching) can be identified (indicated in the Figure 3). The solubilized furosemide complexes are situated in the polymeric chains, thus forming an amorphous solid dispersion.

### 3.3. Morphological Analysis

**Figure 4:**
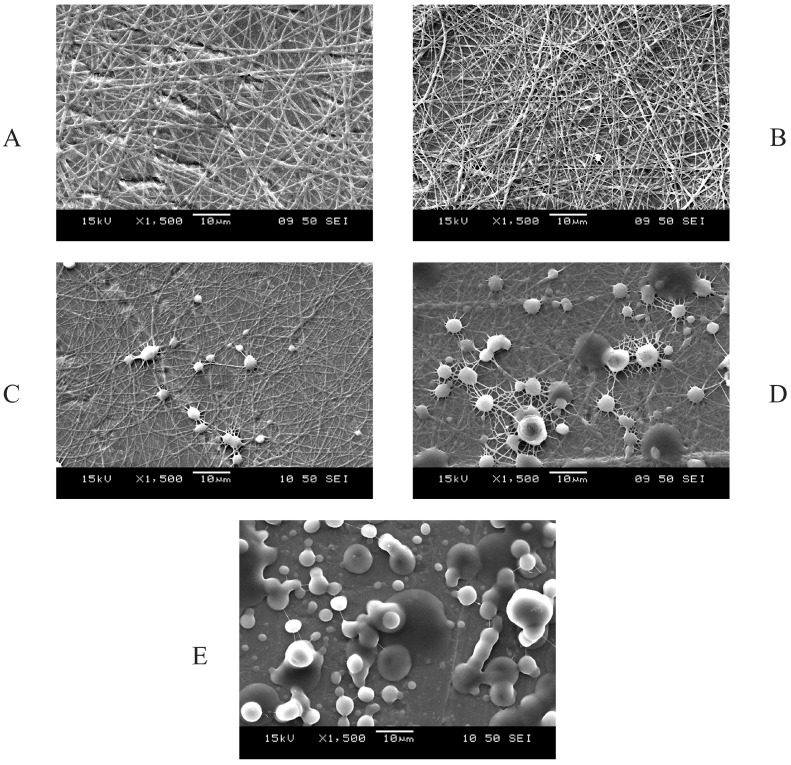
SEM images of the fibers consisting of different HPC-PVP molar ratios. (**A**) HPC-PVP = 5:5 molar ratio; (**B**) HPC-PVP = 6:4 molar ratio; (**C**) HPC-PVP = 7:3 molar ratio; (**D**) HPC-PVP = 8:2 molar ratio; (**E**) HPC-PVP = 9:1 molar ratio.

Along with the changes of the polymer ratio, the corresponding morphology of the electrospun samples also varied. On the scanning electron microscopic images (Figure 4), nanofibers and spray-dried droplets were distinguished. In case of images A and B, the fibrous structural elements are the most dominant, while on images C–E, droplets and randomly oriented fibers can be found, as well. Along with the increase of the HPC ratio in the composites, more droplets formed in the fibrous mats. Based on the SEM images, sample B (HPC-PVP = 6:4) can be considered the most homogeneous fibrous system.

### 3.4. Statistical Analysis of the Fiber Diameters

**Figure 5:**
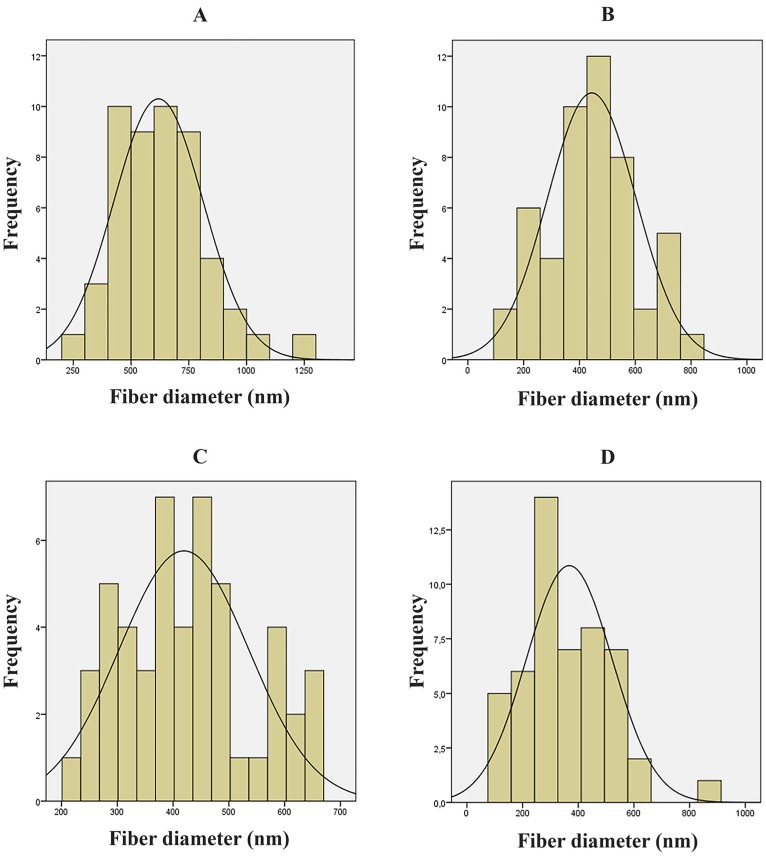
Distribution of fiber diameters. (**A**) HPC-PVP = 5:5 molar ratio; average diameter: 618.76 ± 193.617 nm; (**B**) HPC-PVP = 6:4 molar ratio; average diameter: 443.89 ± 158.376 nm; (**C**) HPC-PVP = 7:3 molar ratio; average diameter: 419.43 ± 116.048 nm; (**D**) HPC-PVP = 8:2 molar ratio; average diameter: 367.50 ± 153.908 nm.

For the statistical analysis of the fiber diameters, only the fibrous samples of HPC-PVP = 5:5; 6:4; 7:3; 8:2 molar ratios were investigated. In the case of HPC-PVP = 9:1 ratio, the diameter distribution cannot be determined, since almost totally electrosprayed structure was formed. The distributions of the four samples were tested using the Kolmogorov–Smirnov test. In all four cases, normality was confirmed (the corresponding *p* values are 0.964; 0.992; 0.641; 0.410). The difference of the sample distributions was analyzed by variance, and significant differences found (*p* < 0.001). Further, pairwise comparisons using the Bonferroni and Scheffe post hoc tests were conducted. The first sample (HPC-PVP = 5:5) shows significant difference from the other three (*p* < 0.001); the other samples are all normally distributed compared to each other. The results of the statistical analysis are illustrated in the Figure 5. The statistical evaluation confirmed that a sample of HPC-PVP = 6:4 ratio is the principally suitable concentration for electrospinning under given preparation conditions, which suggests that in case of the optimum concentration, the characteristics of the fibers are more predictable than in other concentrations.

## 4. Conclusions

Furosemide loaded HPC-PVP composite nanofibrous systems were prepared successfully by electrospinning process. The optimum composition of HPC-PVP fibers was determined based on the rheological examination of polymeric solutions and the scanning electron microscopic morphological characterization of the corresponding electrospun samples. This formulation could enable the transmucosal amorphous drug delivery of the poorly soluble and permeable drug (BCS IV), thus improving the oral bioavailability of furosemide, meanwhile, avoiding its first pass metabolism.
